# Structural, Magnetic, Dielectric and Piezoelectric Properties of Multiferroic PbTi_1−x_Fe_x_O_3−δ_ Ceramics

**DOI:** 10.3390/ma14040927

**Published:** 2021-02-16

**Authors:** Khiat Abd Elmadjid, Felicia Gheorghiu, Mokhtar Zerdali, Ina Turcan, Saad Hamzaoui

**Affiliations:** 1 Laboratoire de Microscopie Electronique & Sciences des Matériaux, Université des Sciences et de Technologie d’Oran (USTO), BP 1505, El M’Naouer, Oran 31000, Algeria; mokhtarzerdali@gmail.com (M.Z.); hamzaoui.saad@gmail.com (S.H.); 2 Research Center in Industrial Technologies (CRTI), P.O. Box 64 Cheraga, Algiers 16014, Algeria; 3 Research Center on Advanced Materials and Technologies, Sciences Department, Institute of Interdisciplinary Research, Alexandru Ioan Cuza University of Iasi, Blvd. Carol I, nr. 11, 700506 Iasi, Romania; 4 Dielectrics, Ferroelectrics & Multiferroics Group, Department of Physics, Alexandru Ioan Cuza University of Iasi, Blvd. Carol I, nr. 11, 700506 Iasi, Romania; ina.turcan@yahoo.com

**Keywords:** dielectric response, Fe-doped PbTiO_3_, magnetic properties, multiferroic ceramics, piezoelectric properties

## Abstract

PbTi_1−x_Fe_x_O_3−δ_ (*x* = 0, 0.3, 0.5, and 0.7) ceramics were prepared using the classical solid-state reaction method. The investigated system presented properties that were derived from composition, microstructure, and oxygen deficiency. The phase investigations indicated that all of the samples were well crystallized, and the formation of a cubic structure with small traces of impurities was promoted, in addition to a tetragonal structure, as Fe^3+^ concentration increased. The scanning electron microscopy (SEM) images for PbTi_1−x_Fe_x_O_3−δ_ ceramics revealed microstructures that were inhomogeneous with an intergranular porosity. The dielectric permittivity increased systematically with Fe^3+^ concentration, increasing up to *x* = 0.7. A complex impedance analysis revealed the presence of multiple semicircles in the spectra, demonstrating a local electrical inhomogeneity due the different microstructures and amounts of oxygen vacancies distributed within the sample. The increase of the substitution with Fe^3+^ ions onto Ti^4+^ sites led to the improvement of the magnetic properties due to the gradual increase in the interactions between Fe^3+^ ions, which were mediated by the presence of oxygen vacancies. The PbTi_1−x_Fe_x_O_3−δ_ became a multifunctional system with reasonable dielectric, piezoelectric, and magnetic characteristics, making it suitable for application in magnetoelectric devices.

## 1. Introduction

Multiferroics are a special class of multifunctional materials that simultaneously exhibit ferromagnetic and ferroelectric properties. In recent years, multiferroic materials have attracted a great interest due to their potential applications in, e.g., spintronics, data-storage media, sensors, transducers, actuators, and multiple-state memories [1,2,3]. The multifunctional character of multiferroic systems is caused by the interaction between electric polarization and spontaneous magnetization, which leads to the most important feature of these materials, which is called the “magnetoelectric (ME) effect”. The coupling between the polarization and magnetization in multiferroics opens the possibility to manipulate the magnetic properties through an electric field and vice versa. This ability results in novel memory devices that use electric and/or magnetic fields for reading/writing operations, thus overcoming the difficulties associated with reading ferroelectric random-access memory [3].

The PbTiO_3_ (PTO) system is a well-known ferroelectric material with a high polar perovskite structure [4,5]. A ferroelectric compound such as PTO is not a ferromagnetic (FM) material due to its lack of a partially filled d-orbital in the Ti^4+^ ions, which results in a diamagnetic behavior. PTO is a well-studied system with different applications, such as humidity sensors [6]. However, the first-principle calculations based on spin-density functional theory suggest that Ti and O vacancy can induce FM characteristics in PTO [7]. Many researchers have reported that oxygen vacancies induce room-temperature ferromagnetism in non-magnetic systems [8,9]. The coexistence of ferroelectricity and ferromagnetism at room temperature in PTO-based systems has a great technological importance. In order to control the induction and enhancement of ferromagnetism while preserving the ferroelectric properties, the strategy of doping PTO with different magnetic transition metals (Fe^3+^, Mn^2+^, etc.) on Ti sites was adopted [10,11,12,13]. Palker et al. [10] reported FM properties and a magneto-electric coupling in Pb(Fe_0.5_Ti_0.5_)O_3_ at room temperature with a saturation magnetization value of *M*_s_ = 0.5 µ_B_/f.u (*M*_s_ = 27 × 10^−3^ emu/g). In Fe-doped PTO nanocrystals, Ren et al. [11] observed room-temperature FM properties with the typical value of *M*_s_ = 0.8 × 10^−3^ emu/g. Such a low value of *M*_s_ was believed to originate from the O vacancy and was explained in terms of the exchange interactions among the Fe^3+^ ions through the trapped electrons in the bridging oxygen ions (F-center). Similar FM properties induced by Fe^3+^–V_O_^2−^–Fe^3+^ networks (F-center exchange mechanism) with a maximum *M*_s_ of 41.6 × 10^−3^ emu/g were reported in Fe-doped PTO nanocrystals [12]. Therefore, the induction of a multiferroic character in a ferroelectric material through suitable doping strategy represents the aim of current research in the field of multifunctional materials. The improvement of Fe-doped PbTiO_3_ is necessary in order to obtain properties required for its use in multifunctional device applications.

The aim of the present work was to study the multifunctional properties of PbTi_1−x_Fe_x_O_3−δ_ ceramics through the solid-state reaction method. In general, doping with Fe^3+^ magnetic ions on PTO leads to the creation of oxygen vacancies depending on the charge compensation, and this is expected to improve the magnetic and ferro-/piezoelectric properties. Experimental investigations of the piezoelectric and dielectric response of Fe-doped PbTiO_3_ are not often reported, and this is one of the goals of the present article. An improvement in both the dielectric and magnetic properties was observed. The correlation between oxygen vacancies and the influence of Fe on the structural and multifunctional (dielectric, piezoelectric, and magnetic) properties is discussed in detail.

## 2. Experimental Details

**Figure 1:**
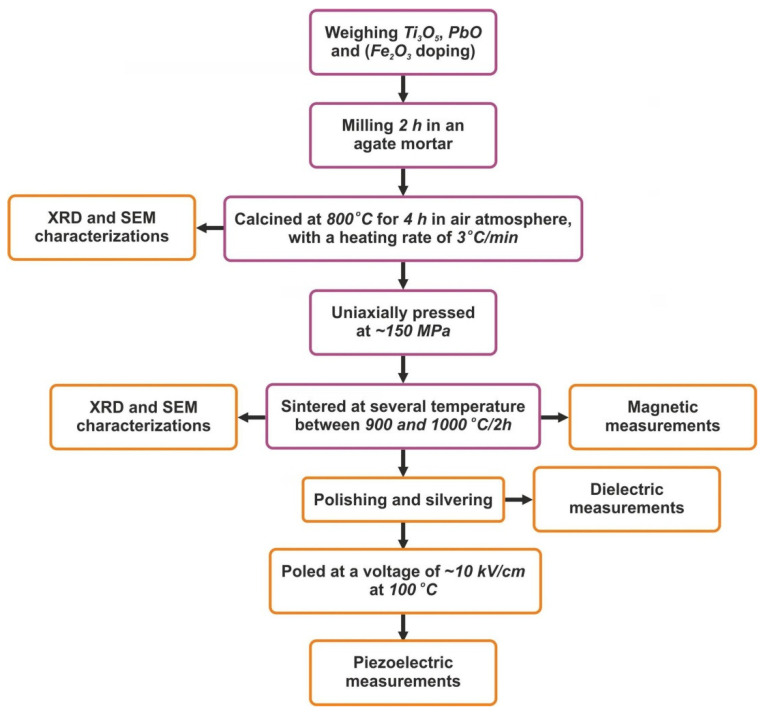
Flowchart of the preparation and investigations of the PbTi_1−x_Fe_x_O_3−δ_ ceramics.

PbTi_1−x_Fe_x_O_3−δ_ (*x* = 0, 0.3, 0.5, and 0.7 amounts of Fe^3+^) ceramics were synthesized with the solid-state reaction method according to the schematic diagram presented in Figure 1. The PbTi_1−x_Fe_x_O_3_ powders were prepared from stoichiometric proportions of high-purity oxide nanopowders: PbO (Alfa Aesar, Johnson Matthey GmbH, Germany, purity > 99.9%, average particle size of 3–5 μm), Ti_3_O_5_ (Alfa Aesar, Johnson Matthey GmbH, Germany, purity > 99.9%, average particle size between 10 and 200 μm), and Fe_2_O_3_ (Alfa Aesar, Johnson Matthey GmbH, Germany, >99.9% purity). The weighted powders were mixed well in an agate mortar for 2 h using ethanol. The resulting powders were calcined at 800 °C for 4 h in air with a heating rate of 3 °C/min. After the calcination step, the resulting powders were reground and pressed into disc-shaped pellets (thickness ~2–3 mm, diameters of ~10 mm) using uniaxial pressing at ~150 MPa, and were then sintered at several temperatures between 900 and 1100 °C for 2 h. The processing parameters (such as heat treatment temperatures and reaction times) were optimized after a series of experiments in order to obtain high purities and densities over 90%.

Phase identification and structural characterization were carried out through X-ray diffraction using a PANalytical Empyrean diffractometer (PANalytical, Almelo, Netherlands) with CuKα radiation (λ = 1.5406 Å) and with scan step increments of 0.04° for 2θ between 20° and 60°. The identification of the phase was performed using the HighScore database software (version 4.9). The microstructural images of the fractured surfaces of these ceramics were obtained using scanning electron microscopy (SEM, type JEOL JCM-5000 Neoscope, Akishima, Tokyo, Japan). The Image J program was employed to estimate the particle size of the samples. The magnetic properties at room temperature were determined under magnetic fields of up to 14 kOe with a vibrating sample magnetometer (VSM, MICROSENSE EV9, Tempe, AZ, USA). Complex impedance measurements at room temperature in the frequency range of 1–10^6^ Hz were performed by using an impedance analyzer (Solartron 1260A Impedance Analyzer, Derby Road, UK) on an Ag-electroded ceramic pellet. For the piezoelectric measurements, the samples were poled at a voltage of ~10 kV/cm generated by a suitable supply in a tube furnace (Nabertherm, mod. L5/13/P330, Lilienthal, Germany) under atmosphere at 100 °C. The applied field was kept during the sample’s cooling. The resonance (*f*_r_) and anti-resonance (*f*_a_) frequencies from which the electromechanical parameters were estimated were obtained using a network analyzer (Agilent technologies E5071C, Santa Clara, CA, USA) with an operating frequency range of 1–50 MHz.

## 3. Results and Discussion

### 3.1. Phase and Structural Characterizations

**Figure 2:**
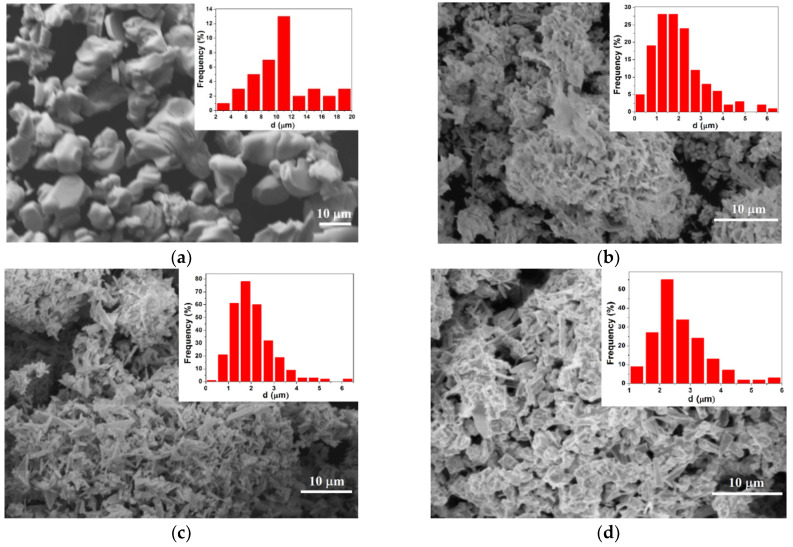
Scanning electron microscopy (SEM) images of the PbTi_1−x_Fe_x_O_3−δ_ powders calcined at 800 °C for 4 h: (**a**) *x* = 0; (**b**) *x* = 0.3; (**c**) *x* = 0.5; and (**d**) *x* = 0.7. Inset: corresponding grain size distributions.

As the particle size of calcined powders is important for the formation of ceramics and their functional properties, the resulting powders were subjected to a scanning electron microscopy analysis. Figure 2 shows the SEM microstructures of the obtained calcined powders. It is observed that with the increase in the Fe dopant, the PbTi_1−x_Fe_x_O_3−δ_ powder microstructure shows various types of particle sizes and shapes. According to Figure 2a, the morphology of the pure PTO particles is that of large particles with average sizes of around 11 µm. Figure 2b–e show the SEM images for Fe-doped PTO powder, which presented a microstructure that consisted of irregular sheet shapes with lateral sizes of around 2 µm. A tendency of particle growth inhibition in the PbTi_1−x_Fe_x_O_3−δ_ powders can be observed, which can be attributed to the crystal lattice distortion originating from the replacement of smaller valence Fe^3+^ ions in larger valence Ti^4+^ sites in the PTO matrix lattice [13].

**Figure 3:**
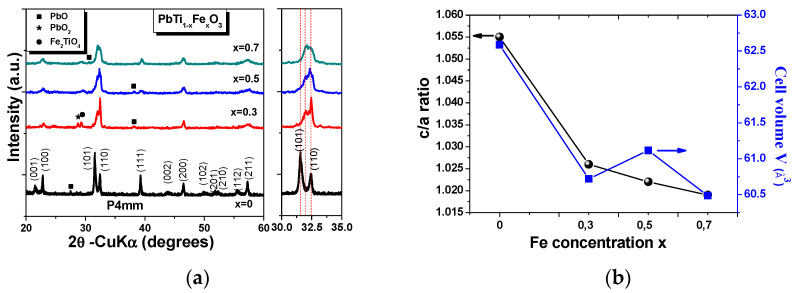
(**a**) Room-temperature X-ray diffraction (XRD) patterns of Fe-doped PbTiO_3_ ceramics. (**b**) Variations in c/a ratio and cell volume with increases in Fe content in PbTiO_3_.

Figure 3a shows the X-ray diffraction (XRD) patterns of PbTi_1−x_Fe_x_O_3−δ_ ceramics, which exhibit sharp diffraction peaks, indicating that all of the samples were well crystallized. In pure PbTiO_3_, the splitting of the (001)/(100) and (101)/(110) peaks revealed the formation of the pure tetragonal phase with the lattice parameters *a* = 3.9 Å and *c* = 4.11 Å, according to the JCPDS Card No. 01-071-4813 [14]. In the case of the PbFe_x_Ti_1−x_O_3_ compositions, the overlapping of the two tetragonal reflections at (101) and (110) and the disappearance of the reflections corresponding to the (102), (201), and (210) planes can be explained by the increase in the “*a*” lattice parameter as Fe^3+^ ion concentration was increased for substitution at Ti^4+^ sites [11]. High concentrations of Fe^3+^ ions affected the tetragonal structural and promoted the formation of a cubic structure in addition to the tetragonal one, which was demonstrated by changes in the peak amplitudes of the diffraction peaks corresponding to the (101/)/(110) planes for the *x* = 0.3 and 0.5 concentrations. For the *x* = 0.7 Fe concentration, the peak amplitudes were almost the same, and this seems to be due to the coexistence of the tetragonal and cubic phases. Moreover, when Fe^3+^ ions substituted onto Ti^4+^ sites, there was a shift of the peak indexed as the (101) plane. When Fe^3+^ was substituted onto Ti^4+^ sites, small traces of impurities, such as PbO, PbO_2_, and Fe_2_TiO_4_, were obtained. It could be observed that the PbO peak from the pure PTO was not visible for the Fe-doped PTO ceramics, which can be explained by the transformation in the PbO_2_ phase. The unit cell parameters, tetragonality (*c/a*), unit cell volume, and crystallite size of the PbTi_1−x_Fe_x_O_3−δ_ system are listed in Table 1. As Fe concentration increased (Figure 3b), the lattice parameter “*a*” almost remained the same, while the “*c*” parameter decreased from 4.114 to 3.975 Å for *x* = 0 and 0.7, respectively. Consequently, the tetragonality (c/a) decreased from 1.055 to 1.019.

**Figure 4:**
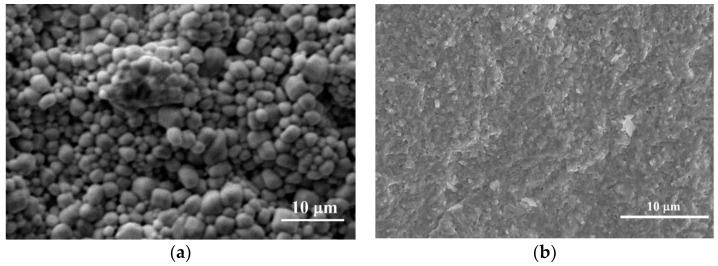

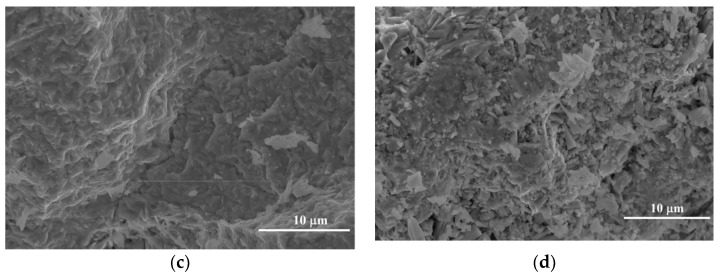
SEM images of the fracture cross-section of PbTi_1−x_Fe_x_O_3−__δ_ ceramics: (**a**) *x* = 0; (**b**) *x* = 0.3; (**c**) *x* = 0.5; and (**d**) *x* = 0.7.

Figure 4 shows the SEM images of the freshly fractured surface of the PbTi_1−x_Fe_x_O_3−__δ_ ceramics. It can be observed that the ceramics present similar microstructures to those of the calcined powder. The SEM images of the pure PTO ceramic show an inhomogeneous morphology, with larger and more agglomerated grains with average sizes of about ~2–3 μm. The Fe-doped PTO microstructures were inhomogeneous and had an intergranular porosity. For the *x* = 0.3 concentration of Fe^3+^ ions, the SEM images show a quite homogeneous microstructure, with small grains of 0.5–1 μm. When the concentration of Fe^3+^ ion addition was increased, the microstructure presented a combined morphology consisting of small faceted grains of 1 μm and plate-like grains. This morphology was more evident with the continuous increase in Fe concentration to *x* = 0.7, for which it can be observed that a large majority of formations were elongated platelets. 

### 3.2. Magnetic Properties 

**Figure 5:**
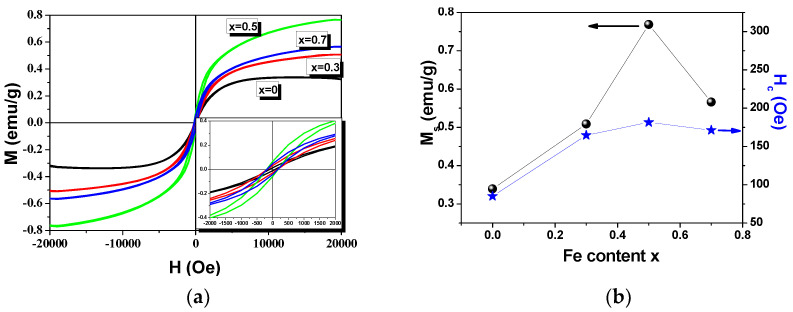
(**a**) Hysteresis loops (M(H)) measured at room temperature for the PbTi_1-x_Fe_x_O_3-δ_ ceramics with different compositions and (**b**) saturation magnetization and magnetic coercivity vs. Fe content.

The room-temperature magnetic properties for the PbTi_1−x_Fe_x_O_3−__δ_ ceramics are shown in Figure 5. A comparison of magnetizations and magnetic fields is presented in Figure 5a. All of the compositions exhibit typical ferromagnetic hysteresis loops, indicating the presence of an ordered magnetic structure. The weak ferromagnetism observed for the pure PTO is due to the presence of oxygen vacancy defects that are created in the system for charge compensation [5,7,8,9]. The doping with Fe^3+^ magnetic ions induced a ferromagnetic behavior, with the highest magnetization and coercive field for the Fe content of *x* = 0.5. The saturation magnetization (*M*_s_) increased with the addition of Fe^3+^ from *M*_s_ = 0.33 emu/g for *x* = 0 to *M*_s_ = 0.76 emu/g for *x* = 0.5 (Figure 5b, black curve). It can also be seen that the magnetic coercivity (*H*_c_) increased with increasing Fe content (Figure 5b, blue curve) from values of *H*_c_ = 85 Oe for *x* = 0 to *H*_c_ = 181 Oe for *x* = 0.5. When the concentration of magnetic ions increased, the ferromagnetism was expected to increase due to the gradual increase in interactions between Fe^3+^ ions mediated by the presence of oxygen vacancies (Fe^3+^–_O_^2-^–Fe^3+^, F–center exchange mechanism (FCE)) [15,16,17]. In the FCE, an electron trapped in the oxygen vacancies constitutes an *F*-center and occupies an orbital that overlaps the *d*-shells of both iron neighbors [17]. It is worth mentioning here that an increase in Fe^3+^ concentration for substitution at Ti^4+^ sites will result in an increase in oxygen vacancies in order to restore the electrical neutrality. Thus, the gradual increase in magnetization can also be ascribed to the increase in the number of oxygen vacancy defects, which are responsible for the room-temperature ferromagnetism. An exception from this increase in *M*_s_ and *H*_c_ was observed in the case of addition of Fe^3+^ at a concentration of *x* = 0.7, for which the increasing number of oxygen vacancies was compensated by the coarsening of the ceramic grain, which reduced the surface ferromagnetism and the porosity presence [18]. It was already proved that the magnetization of nanosized magnetic particles increases with the reduction of particle size [19]. Thus, the improvement of the ferromagnetic properties was also due to the smaller grain sizes of the Fe-doped PTO samples in comparison with the pure PTO ceramic. Hence, multiple factors can affect the magnetic properties, and a detailed magnetic investigation is beyond the scope of the present work.

### 3.3. Dielectric Properties

**Figure 6:**
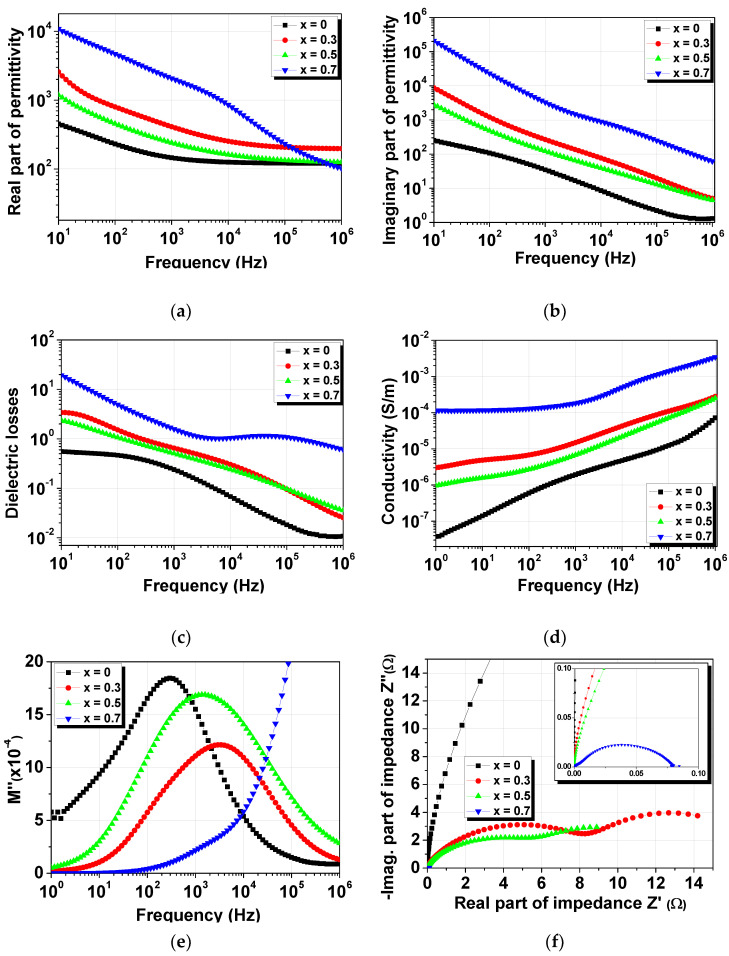
Dielectric properties vs. frequency measured at room temperature for PbTi_1−x_Fe_x_O_3−δ_ ceramics: (**a**) real part of the permittivity, (**b**) imaginary part of the permittivity, (**c**) tangent loss, (**d**) conductivity, (**e**) imaginary part of the dielectric modulus, and (**f**) complex impedance spectra.

Figure 6a–f show the room-temperature dielectric properties measured as a function of frequency in the range 10 Hz–1 MHz. Very high values of permittivity in the range 1000–12,000 were observed at low frequencies (< 10^2^ Hz) for all of the compositions (Figure 6a), followed by a monotonous decrease with frequency. The high values of the real part of the permittivity in the low-frequency range can be due to the Maxwell–Wagner phenomena that are caused by the presence of oxygen vacancies in the PbTi_1−x_Fe_x_O_3−δ_ ceramics, which are formed as a means of charge compensation when Fe^3+^ replaces Ti^4+^ in the unit cells of perovskite. In addition, the substitution of Fe^3+^ ions onto the Ti^4+^ sites leads to a systematic increase in permittivity, except at high frequencies (>10^5^ Hz), where the permittivity for the *x* = 0.7 concentration strongly decreases. This can be explained by taking into account the structural modifications (the coexistence of tetragonal and cubic phases), which lead to the diminishing of the ferroelectric character. The room-temperature dependences of the dielectric losses vs. frequency are presented in Figure 6c. It can be observed that the losses also diminish with increasing frequency for all compositions, and the ceramics with Fe^3+^ concentrations up to *x* = 0.5 show values above unity for high frequencies over 10^5^ Hz. The almost constant values of the real and imaginary parts (Figure 6b) of the permittivity at high frequencies, accompanied by the small values of the dielectric losses, can be considered intrinsic values that characterize the PbTi_1−x_Fe_x_O_3−δ_ system. However, losses above unity, in addition to the high values of permittivity in the low-frequency range, indicate important contributions from the combination of conductivity behavior with Maxwell–Wagner phenomena to the total dielectric response. The differences in the complex dielectric behaviors of the PbTi_1−x_Fe_x_O_3−δ_ ceramics can be due to the different types of microstructures, the presence of secondary phases, and the different concentrations of oxygen vacancies induced by the increase in Fe^3+^ concentration or/and during sintering.

Figure 6d shows the comparative frequency dependence of the ac-conductivity measured at room temperature. The behavior of the ac-conductivity of the PbTi_1−x_Fe_x_O_3−δ_ ceramics exhibited different regions in the studied frequency range, which suggests that there was more than one mechanism that contributed to the ceramics’ conduction behavior: one mechanism that corresponds to the grain boundaries’ contributions through the hopping of carrier charges, which is attributed to the low-frequency range, and another mechanism associated with the monotonous increase in conductivity in the high-frequency region due to the grain cores’ contributions.

The dielectric modulus formalism combined with complex permittivity analysis can be used in order to understand if a conduction mechanism or multiple dielectric relaxations are responsible for the complex dielectric response of the PbTi_1−x_Fe_x_O_3−δ_ system. The complex dielectric modulus is defined as in the following equation:

(1) $$M^{*}\left(f\right)=\frac{1}{\varepsilon^{*}\left(f\right)}=M^{\prime }\left(f\right)+iM^{\prime \prime }\left(f\right)$$

The real and imaginary parts of the dielectric modulus are given by the following formulas:

(2) $$M^{\prime }\left(f\right)=\frac{\varepsilon^{\prime }\left(f\right)}{\varepsilon^{\prime }{}^{2}\left(f\right)+\varepsilon^{\prime \prime }{}^{2}\left(f\right)},\;\;M^{\prime \prime }\left(f\right)=\frac{\varepsilon^{\prime \prime }\left(f\right)}{\varepsilon^{\prime }{}^{2}\left(f\right)+\varepsilon^{\prime \prime }{}^{2}\left(f\right)}$$

Figure 6e shows the imaginary part of the dielectric modulus measured at room temperature for the PbTi_1−x_Fe_x_O_3−δ_ ceramics. All of the ceramics apparently presented only one broad peak, which was shifted toward higher frequencies as Fe^3+^ concentration increased. The presence of a peak in *M″(f)* and the lack of peak in *ε″(f)* confirm the conductivity relaxation mechanism’s contribution to the total dielectric response.

Figure 6f shows the complex impedance spectra measured at room temperature for the PbTi_1−x_Fe_x_O_3−δ_ ceramics. While the pure PbTiO_3_ ceramic presented a single component of the Z″(Z′) dependence, the Fe-doped PTO ceramics presented more than one semicircle, demonstrating a local electrical inhomogeneity. In a ceramic, it is impossible to control the local oxygen stoichiometry [20], and this causes local electrical inhomogeneities in the sample volume. A single semicircle in the PTO complex spectra demonstrates a good dielectric and conductive homogeneity within the sample [5]. In general, the dielectric properties of a polycrystalline ceramic are due to the ceramic grain, ceramic grain boundary regions, and electrode–ceramic interfaces, which have different dielectric and conductive responses. In this case, the real homogeneous dielectric properties can be described by an resistor-capacitor (RC)-equivalent circuit [21,22]. In the Fe-doped PTO complex spectra, the presence of more than one semicircle indicated contributions from the ceramic grain and ceramic grain boundary regions. The difference between the grain bulk’s and grain boundaries’ responses most probably came from the higher number of boundaries (as a consequence of smaller average grain size) that were present in the doped ceramics, different oxygen vacancies that were in a higher concentration at the grain boundaries, and the secondary phases’ presence, which was confirmed by the XRD measurement results.

### 3.4. Piezoelectric Properties

**Figure 7:**
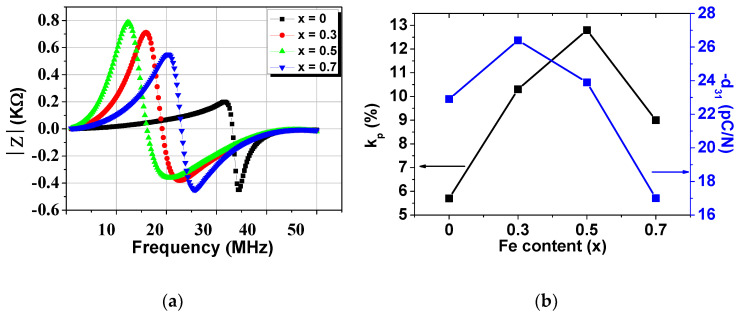

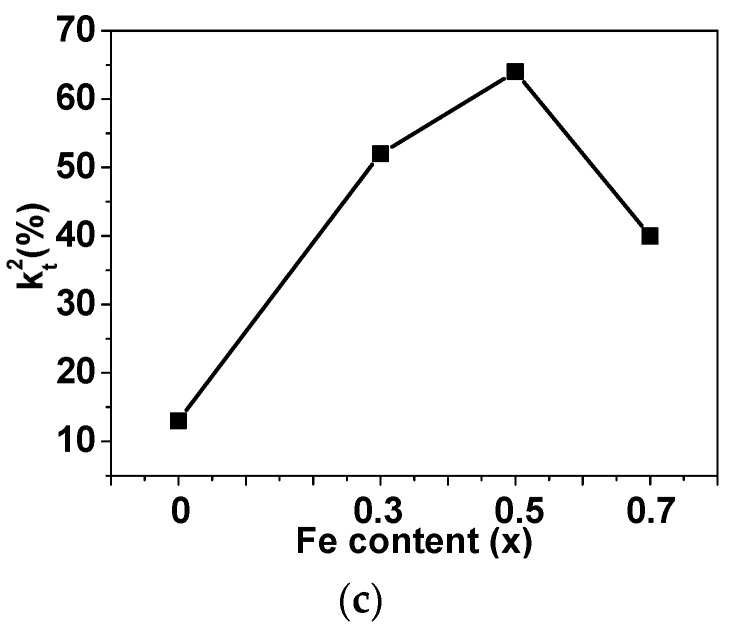
(**a**) Electrical impedance spectra vs. frequency; (**b**) variations in the piezoelectric parameters vs. Fe content: piezoelectric coefficient and the planar electromechanical coupling factor; (**c**) the thickness extensional mode electromechanical coupling factor.

In general, piezoelectric properties can be affected by many parameters, such as phase purity, density, grain size, and poling condition. In this study, all samples presented grain sizes between 2 and 3 μm and relative densities in the range of 6.13–7.14. In Figure 7, the results of the piezoelectric study of the PbTi_1−x_Fe_x_O_3−δ_ ceramics are presented. Figure 7a shows the electrical impedance as a function of frequency, from which the resonance (*f*_r_) and antiresonance (*f*_a_) frequencies were obtained in order to calculate the piezoelectric parameters.

The piezoelectric charge coefficient (*d*_31_), the planar electromechanical coupling factor (*k*_p_), and the thickness extensional mode coupling factor (*k*_t_) were influenced by the substitution of Fe^3+^ ions onto the Ti^4+^ sites of the PbTiO_3_ matrix. The electromechanical coupling factor is the key parameter when describing the behavior of piezoelectric materials due the fact that it represents a numerical measure of efficiency for the electromechanical conversion of the piezoelectric response. For the calculation of these parameters, the following equations were used:

(3) $$k_{p}\approx {\left(\frac{f_{a}^{2}-f_{r}^{2}}{f_{a}^{2}}\right)}^{1/2}$$

(4) $$d_{31\;}{=\;k}_{31}\sqrt{{\varepsilon \;s}_{11}^{E}}$$

(5) $$k_{31}=\sqrt{\frac{\frac{\pi }{2}\frac{f_{a}}{f_{r}}}{\frac{\pi }{2}\frac{f_{a}}{f_{r}}-\tan\left(\frac{\pi }{2}\frac{f_{a}}{f_{r}}\right)}}$$

(6) $$s_{11}^{E}=\frac{1}{4\rho f_{r}^{2}w^{2}}$$

(7) $$k_{t}^{2}=\frac{\pi }{2}\frac{f_{r}}{f_{a}}\tan\left(\frac{\pi }{2}\left(\frac{f_{a}-f_{r}}{f_{a}}\right)\right)$$

where $k_{p}$ $k_{31}$, $s_{11}^{E}$, *w*, and ρ are the planar coupling factor, electromechanical coupling factor in the length-extensional mode, elastic coefficient at a constant electric field, width of the ceramics, and density (in g/cm^3^), respectively. All of the calculated parameters are listed in Table 2.

In Figure 7b, it can be observed that the piezoelectric coefficient presents a maximum for the *x* = 0.3 composition, which also presents the highest relative density; then, the values decreases significantly with increases in Fe concentration. The decrease in the piezoelectric charge coefficient can be explained by taking into account the decrease in tetragonality with increasing Fe content, which affects the ferroelectricity of the system, as well as the moderately low density. In comparison with pure PbTiO_3_ ceramics reported in the literature or with similar perovskite systems, these values are much smaller [23,24]. This result confirms that for a small electric field of 10 kV/cm, a high Fe content does not favor the obtainment of good piezoelectric properties; this result is in agreement with the results of other piezoelectric study of BaTi(_1-x_)Zr_x_O_3_ (BTZ) ceramics [25]. However, the maximum value of *d*_31_ obtained for the highest density corresponded to the Fe concentration of *x* = 0.3, making the PbTi_1−x_Fe_x_O_3−δ_ ceramics suitable candidates for obtaining better piezoelectric performance in the future by tailoring the microstructural properties.

In conclusion, the complex investigations of the substitution of Fe^3+^ ions onto the Ti^4+^ sites of the PbTiO_3_ matrix have shown the coexistence of electrical, moderate piezoelectric, and magnetic properties at room temperature, indicating that PbTi_1−x_Fe_x_O_3−δ_ is a multifunctional system with reasonable dielectric, piezoelectric, and magnetic character, which makes it suitable for application in magnetoelectric devices.

## 4. Conclusions

The main objective of this work was to study the influence of magnetic Fe^3+^ ions on the functional properties of PbTi_1−x_Fe_x_O_3−δ_ ceramics that were prepared through solid-state reactions with amounts of Fe^3+^ in the range *x* = 0 ÷ 0.7. The X-ray analysis results revealed that high concentrations of Fe^3+^ ions affect the tetragonal structural and promote the formation of a cubic structure in addition to the tetragonal one that was observed in the pure PTO. The complex impedance spectra demonstrate that, through Fe substitutions, a local electrical inhomogeneity is induced, as demonstrated by the presence of more than one semicircle in the Z”(Z’) dependencies. The increase in Fe^3+^ ion concentration leads to a systematic increase in permittivity, except for the *x* = 0.7 concentration, for which the dielectric response can be explained by taking the structural modifications into account. By increasing the Fe concentration, a ferromagnetic character is induced at room temperature, with the highest saturation magnetization of *M*_s_ = 0.76 emu/g for an Fe content of *x* = 0.5. The improvement of the magnetic properties can be explained by taking into account the F-center exchange mechanism, where the gradual increase in interactions between Fe^3+^ ions is mediated by the presence of oxygen vacancies. The piezoelectric results confirm that a high Fe content does not favor the obtainment of good piezoelectric properties. Thus, the present study indicates that the substitutions of Fe^3+^ magnetic ions onto Ti^4+^ cation sites make PbTi_1−x_Fe_x_O_3−δ_ a potential multifunctional material for magnetoelectric applications. In the future, further investigations of the newly developed Fe-doped PTO will be carried out in order to obtain more promising properties and, in particular, to investigate the role of Fe in the structural and functional properties of PbTiO_3_.

## Figures and Tables

**Table 1:** The structural results obtained for an Fe-doped PbTiO_3_ (*x* = 0, 0.3, 0.5, 0.7) bulk sample at room temperature.

Samples	Lattice	Parameters	Distortion Ratio	Cell Volume	Crystallite Size
-	*a*(Å)	*c*(Å)	*c/a*	*V* (Å^3^)	*A* (nm)
*x* = 0	3.900	4.114	1.055	62.586	31.1
*x* = 0.3	3.896	3.999	1.026	60.719	8.8
*x* = 0.5	3.910	3.997	1.022	61.117	9.8
*x* = 0.7	3.900	3.975	1.019	60.487	12.5

**Table 2:** The piezoelectric parameters calculated for the poled Fe-doped PbTiO_3_ (*x* = 0, 0.3, 0.5, 0.7) ceramics.

Samples	ρ (g/cm^3^)	*W (mm)*	*f*_r_ (MHz)	*f*_a_ (MHz)	ε	*k*_31_	*k*_p_(%)	$s_{11}^{E}$ (10^−12^ m^2^/N)	−*d*_31_ (pC/N)
*x* = 0	6.34	1.20	32	34	123	4.25	5.7	2.37	22.9
*x* = 0.3	7.14	1.22	16	22	203	2.05	10.3	9.42	26.4
*x* = 0.5	6.13	1.17	12	20	125	1.58	12.8	16.8	23.9
*x* = 0.7	6.44	1.31	20	25	101	2.39	9	6.07	17.0

## Data Availability

Not applicable.
